# Impact of clonal *TP53* mutations with loss of heterozygosity on adjuvant chemotherapy and immunotherapy in gastric cancer

**DOI:** 10.1038/s41416-024-02825-1

**Published:** 2024-08-31

**Authors:** Yun Gu, Mengyao Sun, Hanji Fang, Fei Shao, Chao Lin, Hao Liu, He Li, Hongyong He, Ruochen Li, Jieti Wang, Heng Zhang, Jiejie Xu

**Affiliations:** 1 Department of General Surgery, Shanghai Sixth People’s Hospital Affiliated to Shanghai Jiao Tong University School of Medicine, Shanghai, China; 2 NHC Key Laboratory of Glycoconjugate Research, Department of Biochemistry and Molecular Biology, School of Basic Medical Sciences, Fudan University, Shanghai, China; 3 Department of Gastrointestinal Surgery, Zhongshan Hospital, Fudan University, Shanghai, China; 4 Department of Oncology, Shanghai General Hospital, Shanghai Jiao Tong University School of Medicine, Shanghai, China; 5 Department of Emergency Surgery, Zhongshan Hospital, Fudan University, Shanghai, China; 6 Department of Endoscopy, Fudan University Shanghai Cancer Center, Shanghai, China

**Keywords:** Gastric cancer, Cancer genomics, Cancer microenvironment, Predictive markers

## Abstract

**Background:**

This study aimed to reveal the effect of *TP53* status on clinical outcomes and underlying mechanism in gastric cancer (GC) patients.

**Methods:**

*TP53* status was divided into three groups according to genome sequencing, namely clonal mutations with LOH (C-LOH), clonal diploid or subclonal mutations (CD-SC), and wild type (WT). The p53 protein activity was divided into over-expression (OE), Null and WT according to immunohistochemical staining. Four cohorts, including the TCGA, SMC, ZSHS and FUSCC cohort, were analyzed for association between TP53 mutation status and clinical outcomes and the underlying mechanism.

**Results:**

In TCGA cohort, *TP53* CD-SC were associated with superior overall survival compared to *TP53* C-LOH cases. GC patients could benefit from ACT only in *TP53* CD-SC/ p53 OE and *TP53*/ p53 WT subgroups, and *TP53* C-LOH subgroup demonstrated the worst response to pembrolizumab among three subgroups. Genomic and immunophenotypic deconvolution revealed that *TP53* C-LOH, CD-SC and WT differed for genomic and immune-related features.

**Conclusions:**

*TP53* C-LOH GCs with genomic instability and immune evasion phenotype have poor clinical outcomes in patients treated with ACT or immunotherapy.

## Introduction

*TP53*, located at 17p13.1, encodes the p53 protein, which guards cells against cancerous transition by promoting cell cycle arrest, apoptosis and DNA damage repair [1, 2]. Ordinarily, p53 acts as a transcription factor to govern the expression of target genes, such as *CCNE1* and *ATM* [3]. Furthermore, p53 can exert transcription-independent protective roles through interaction with other transcription factors and cellular compartments pathways [4–7]. In addition to its cell-intrinsic efficacy, p53 can also wheel tumor immune microenvironment (TME) [8]. Large studies have revealed that the p53 controls tumor-immune system crosstalk by modulating cytokines, MHC-I antigen processing pathway, and immune checkpoints [9].

*TP53* mutations occur ~50% in gastric cancer (GC), up to 70% in metastasis, and predominantly in tumors with chromosomal instability (CIN) [10, 11]. However, *TP53* mutations in GC have controversial impact on survival and chemotherapy sensitivity [10]. The initial studies showed that *TP53* mutational status was independent indicator to poor survival and response to cisplatin-based chemotherapy [12–14], which were challenged by later research [15, 16]. Furthermore, mutated *TP53* was found to inhibit gastric cancer immunity [17], and predict poor response to immunotherapy in patients with metastatic solid tumors including GC [18]. Moreover, *TP53* loss of heterozygosity (LOH) indicating a severe p53 dysfunction have been reported in GC, revealing an association with tumor progression and poor patient survival [19–21]. These preceding studies with heterogeneous definition of *TP53* alteration presented limited explanation of mechanism, and failed to reach a consensus on how *TP53* alteration influence clinical outcomes in GC.

Previous studies have confirmed the efficacy of the VAF/TP clinical classifier in characterizing gene mutation statuses and differentiating between genomic changes and clinically neutral events [22]. We divided *TP53* status into three groups according to genome sequencing, namely, clonal mutations with LOH (C-LOH), clonal diploid or subclonal mutations (CD-SC), and wild type (WT). Likewise, according to immunohistochemical (IHC) staining, p53 expression was divided into over-expression (OE), Null and WT. Our study, including the Cancer Genome Atlas (TCGA) cohort, the Samsung Medical Center (SMC) cohort, the Zhongshan Hospital (ZSHS) cohort and the FUSCC cohort, studied the association between *TP53* status and clinical outcomes (survival, chemotherapy and immunotherapy sensitivity), and evaluated the underlying mechanism (genomes and TME).

## Patients and methods

### Study population

Overall 890 patients from four cohorts were enrolled in this study, including the ZSHS cohort based on tissue samples, two cohorts based on sequencing data (namely the TCGA and SMC cohorts), and the FUSCC cohort, which possesses both IHC staining data and targeted-genes sequencing data. The ZSHS Cohort originally enrolled 496 GC patients with full informed consent and the approval of the Clinical Research Ethics Committee of Zhongshan Hospital affiliated with Fudan University (Shanghai, China; approval number: Y2015-054). All these patients underwent partial or total gastrectomy in Zhongshan Hospital between August 2007 and December 2008. However, 23 patients were excluded due to dot loss or incomplete clinical information, and we ultimately included 473 patients from Zhongshan Hospital in this study. These patients were followed until April 2014 with a 42-month median follow-up time. The FUSCC cohort included 27 GC patients with full informed consent and the study was approved by the Institutional Review Board (IRB) of FUSCC (IRB number 050432-4-2108). All the patients underwent gastrectomy and targeted-genes sequencing in Fudan University Shanghai Cancer Center during January 2022 to August 2023. Specifically, we included TCGA cohort (*n* = 410) as the discovery cohort to measure *TP53* mutation status; similarly, ZSHS cohort (*n* = 473) was included as the validation cohort to assess p53 protein expression patterns. FUSCC cohort (*n* = 27) was included to confirm the consistency of *TP53* mutation status with p53 protein expression. SMC cohort (*n* = 43) was included to explore the predictive value of *TP53* mutation status for responsiveness to anti-PD-1 immunotherapy in GC patients.

### Immunohistochemistry (IHC)

Firstly, the tissue microarray slides were baked at 60 °C for 6 h, placed in xylene and gradient alcohol to dewax, and then washed with phosphate-buffered saline for 3 times. To inhibit endogenous peroxidase, we treated the slides with 3% H_2_O_2_ for 30 min at 37 °C. Next, the slides were performed heat mediated antigen retrieval with 0.01 M citrate buffer (pH 6.0). After blocked with 10% goat serum (ZSGB-BIO) at 37 °C for 2 h for eliminating non-specific reactions, the slides were subsequently incubated with prediluted primary anti-p53 antibody (Leica, NCL-L-p53-DO7, 1: 800) at 4 °C overnight. Subsequent to reaction with HRP-labeled secondary antibody (ZSGB-BIO) for 30 min at 37 °C, diaminobenzidine (DAB)-H_2_O_2_ and hematoxylin were applied to stain the reaction products and nucleus, respectively. Finally, the slides were fixed with mounting medium for analysis.

### Evaluation of IHC score

IHC images were evaluated by two experienced pathologists under a microscope at high power field (HPF, ×200), both of whom were blinded to the patients’ clinical data. Representative images of IHC staining and corresponding Hematoxylin and Eosin staining were shown in Supplementary Fig. S1. Cases were assigned to one of three categories: (1) WT: 1–80% of tumor cell nuclei staining positive, usually with variable intensity; (2) Null: no tumor cell nuclear staining with a positive internal control; and (3) OE: uniform and intense nuclear staining in at least 80% of tumor cell nuclei (estimated). As for tumors with contradictory disposition in p53 expression pattern from the two pathologists, a second-round evaluation would be conducted.

### Computational analysis

Statistical analyses included in this study were performed by SPSS (version 26.0) and R software (version 4.1.2). All conducted analyses were two sided, and *P* < 0.05 was considered statistically significant. Continuous variables were analyzed by student’s *t* test or one-way ANOVA followed by Tukey’s multiple comparisons. For categorical variables, Chi-square test was adopted. The cut-off value of variant allele frequency (VAF) / tumor purity (TP) for the classification of *TP53* C-LOH and *TP53* CD-SC subgroups was set to 0.9. For the SMC cohort, 40 differentially expressed genes were utilized to generate the classification of *TP53* C-LOH and *TP53* CD-SC based on transcriptomic profiling, with the relevant genes being listed in Supplementary Table S1. The genomic characteristics of the patients from TCGA, containing VAF and TP data, fraction of genome altered (FGA), aneuploidy score and whole-genome doubling (WGD), were directly downloaded from cBioPortal (http://www.cbioportal.org). The cut-off value of Homologous Recombination Deficiency (HRD) score for the classification of HRD and Homologous Recombination Proficiency (HRP) subgroups was set to 42. Associations between *TP53* mutation status and enrichment of immune-related signatures (IFN-α Response, IFN-γ Response, Antigen Processing and Presentation) in the TCGA cohort were assessed with gene sets enrichment analysis (GSEA) using the GSEA software. The immune-related transcription factors activities including *STAT5B*, *STAT5A*, *STAT2*, *IRF1*, *STAT3*, *REL*, *NFKB1*, *RELA*, and *STAT1* were assessed with DoRothEA. Other immune-related features were directly acquired from GDC pan-immune data portal (https://gdc.cancer.gov/about-data/publications/panimmune).

## Results

### *TP53* CD-SC associates with superior overall survival in gastric cancer

**Fig. 1:**
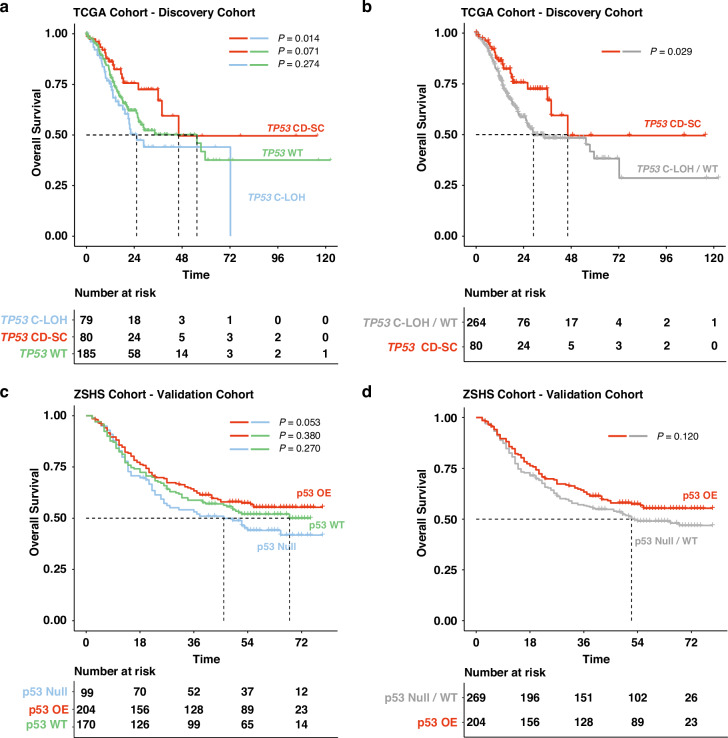
Clinical outcomes of *TP53*/p53-based subgroups in the TCGA and ZSHS cohort. Kaplan-Meier survival curves for the three subgroups of interest (*TP53* C-LOH, *TP53* CD-SC and *TP53* WT) in the TCGA cohort (**a**). Kaplan-Meier survival curves for comparing *TP53* C-LOH with other tumors in the TCGA cohort (**b**). Kaplan-Meier survival curves of the three subgroups of interest (p53 OE, p53 Null and p53 WT) in the ZSHS cohort (**c**). Kaplan-Meier survival curves for comparing p53 OE with other tumors in the ZSHS cohort (**d**). CD-SC, clonal diploid-subclonal; C-LOH, clonal with loss of heterozygosity; WT wild type; OE overexpression. Log-rank test was conducted for Kaplan-Meier curves. *P* ≤ 0.05 was considered statistical significance.

To explore the impact of *TP53* mutation status on prognosis of gastric cancer patients, we conceived variant allele VAF / TP value to classify *TP53* mutations as *TP53* C-LOH, *TP53* CD-SC, and *TP53* WT in the TCGA cohort (Discovery Cohort). The Kaplan-Meier curve showed that patients with *TP53* CD-SC had superior overall survival (OS) compared with *TP53* C-LOH patients (log rank *P* = 0.014, Fig. 1A). A similar trend was also found between the OS of *TP53* CD-SC and *TP53* C-LOH subgroup (log rank *P* = 0.071, Fig. 1A), despite no statistical significance. Thus, we combined *TP53* C-LOH with *TP53* WT subgroup for further analysis and found a significant longer OS in patients with *TP53* CD-SC compared with other patients (log rank *P* = 0.029, Fig. 1B). To understand the prognostic effects of p53 proteins in GC, we divided patients into p53 WT, p53 OE or p53 Null subgroups according to IHC staining in ZSHS cohort (Validation Cohort). However, although a similar trend to that in the TCGA cohort was observed between p53 null vs p53 OE (log rank *P* = 0.053, Fig. 1C), there was no significant difference in OS among p53 OE vs p53 Null/WT (log rank *P* = 0.120, Fig. 1D). To unify the results of the TCGA and ZSHS cohorts, we evaluated the correlation between *TP53* status and p53 expression in the FUSCC cohort. As showed in Supplementary Fig. S2, *TP53* C-LOH and CD-SC consistently exhibited high correlation with p53 Null and OE, respectively. Furthermore, 11/13 patients with *TP53* WT displayed p53 WT expression, while 2/13 showed p53 OE, indicating a consistent relationship between *TP53* status and p53 expression patterns.

### *TP53* C-LOH predicts worse adjuvant chemotherapy response in gastric cancer

**Fig. 2:**
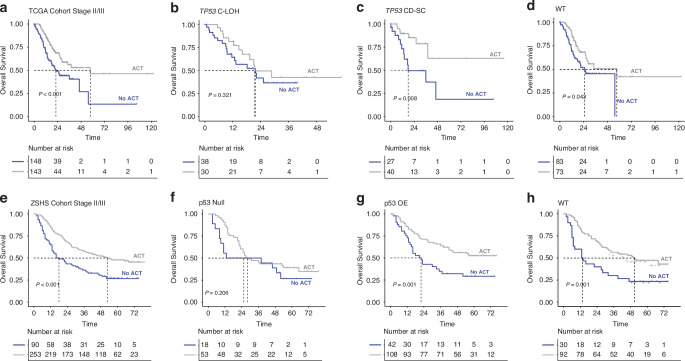
Benefits from adjuvant chemotherapy in patients with different *TP53*/p53 status. Kaplan-Meier analyses of overall survival according to ACT chemotherapy treatment for all stage II/III gastric cancer patients (**a**), *TP53* C-LOH subgroup (**b**), *TP53* CD-SC subgroup (**c**) and *TP53* WT subgroup (**d**) in the TCGA cohort. Kaplan-Meier analyses of overall survival according to ACT chemotherapy treatment for all stage II/III gastric cancer patients (**e**), p53 Null subgroup (**f**), p53 OE subgroup (**g**) and p53 WT subgroup (**h**) in the ZSHS cohort. CD-SC, clonal diploid-subclonal; C-LOH, clonal with loss of heterozygosity; WT wild type; OE overexpression. Log-rank test was conducted for Kaplan-Meier curves. *P* ≤ 0.05 was considered statistical significance.

It has been widely recognized that treatment with fluorouracil-based adjuvant chemotherapy (ACT) could improve the OS for patients with stage II and III gastric cancer [23, 24], which was also identified in the TCGA and ZSHS cohort (log rank *P* < 0.001 and log rank *P* < 0.001, respectively; Fig. 2a, e). Considering the important role of *TP53* gene in chemotherapy response [25], we further explored the association between ACT and OS among patients with stage II/III disease belonging to different *TP53*-based subgroups. Interestingly, fluorouracil-based ACT could only improve overall survival in *TP53* CD-SC (log rank *P* = 0.008, Fig. 2c) and *TP53* WT (log rank *P* = 0.043, Fig. 2d) subgroup, but no longer improved overall survival in patients with *TP53* C-LOH (log rank *P* = 0.321, Fig. 2b). While in ZSHS cohort, we found that ACT had a positive prognostic effect only in p53 OE (log rank *P* = 0.001, Fig. 2g) and p53 WT (log rank *P* = 0.001, Fig. 2h) subgroups, but not in p53 Null subgroup (log rank *P* = 0.206, Fig. 2f).

### *TP53* C-LOH predicts worse anti-PD1 immunotherapy response in gastric cancer

**Fig. 3:**
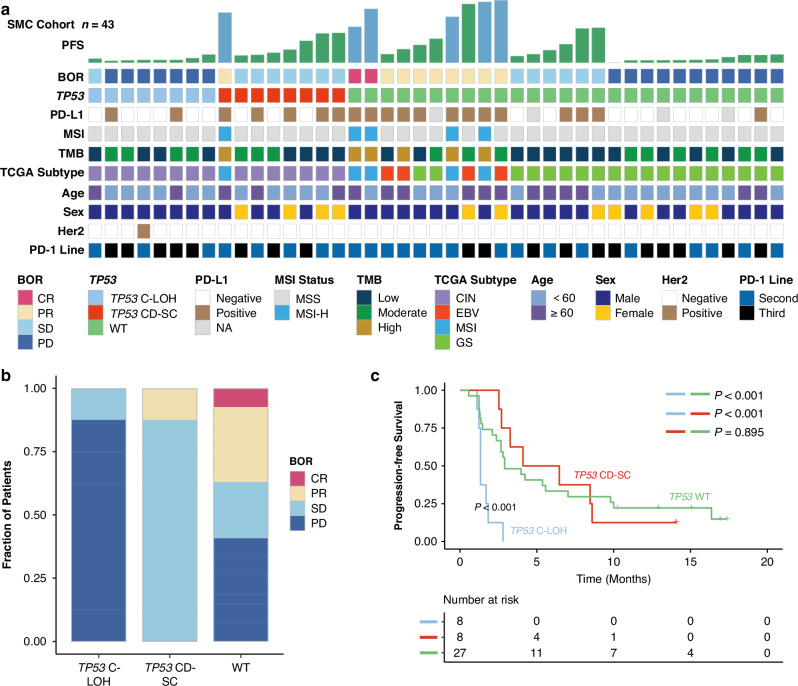
*TP53* status predicts responsiveness to anti-PD-1 immunotherapy in gastric cancer. Heatmap demonstrated responsiveness to pembrolizumab and molecular parameters based on *TP53* status in the SMC cohort (*n* = 43) (**a**). Association of the BOR with the *TP53* status in the ICB cohort (**b**). Kaplan-Meier curves of overall survival based on *TP53* status in ICB cohort (*n* = 43) (**c**). BOR best of response, CIN chromosomal instability, EBV EBV-positive, GS genomically stable, MSI microsatellite instability, TMB tumor mutation burden. Log-rank test was conducted for Kaplan-Meier curves. *P* ≤ 0.05 was considered statistical significance.

Recently, immunotherapy have been playing more and more important roles in GC treatment. We further enrolled another cohort from the Samsung Medical Center (the SMC cohort) consisting of patients treated with anti-PD1 immunotherapy to evaluate the predictive value of *TP53* status for immunotherapy. The baseline clinical and molecular characteristics of the SMC cohort were displayed in Fig. 3a. We found that patients in *TP53* C-LOH subgroup demonstrated a significantly decreased response rate compared with those in *TP53* CD-SC and *TP53* WT subgroup (*P* < 0.001, Fig. 3b). Meanwhile, patients in *TP53* C-LOH subgroup demonstrated the worst OS among all patients treated with anti-PD1 immunotherapy (log rank *P* < 0.001, Fig. 3c), and there was no significant overlap between *TP53* mutation status and existing immunotherapy response predictive markers [26], such as microsatellite instability status and PD-L1 expression (Supplementary Fig. S3).

### Correlation between genomic feature and *TP53* status in gastric cancer

**Fig. 4:**
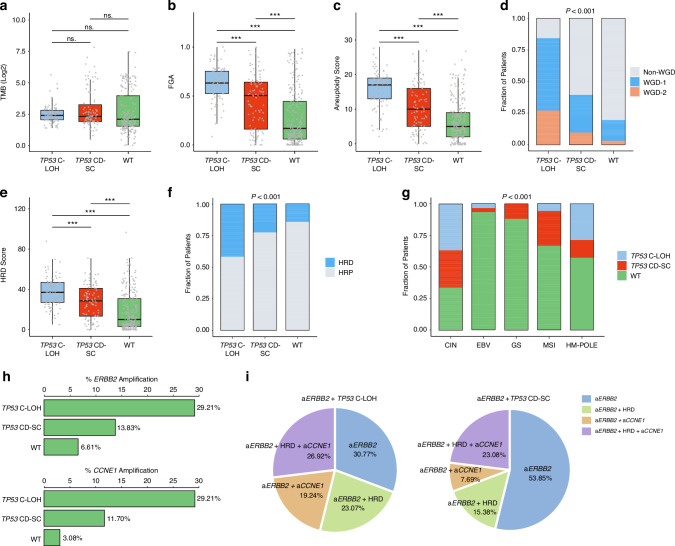
Genomic characteristics associated with different *TP53* status in gastric cancer. Box plots for TMB (**a**), FGA (**b**), aneuploidy score (**c**) in the three subgroups of interest (*TP53* C-LOH, *TP53* CD-SC and *TP53* WT). Stacked bar chart displaying the distribution of WGD across the three subsets of interest (*TP53* C-LOH, *TP53* CD-SC and *TP53* WT) (**d**). Box plots for HRD score in the three subgroups of interest (*TP53* C-LOH, *TP53* CD-SC and *TP53* WT) (**e**). Stacked bar chart displaying the association of HRR status (**f**) and TCGA subtypes (**g**) with *TP53* status. Analyses of ERBB2 and CCNE1 copy number alteration in the three subgroups of interest (*TP53* C-LOH, *TP53* CD-SC and *TP53* WT) (**h**). Different genomic alterations accompanied with *ERBB2* amplification in *TP53* C-LOH and *TP53* CD-SC subgroups (**i**). TMB tumor mutation burden, FGA fragment genome alteration, WGD whole genome duplication. CD-SC clonal diploid-subclonal, C-LOH clonal with loss of heterozygosity, WT wild type.

Given the comprehensive impact on therapeutic responsiveness, we next tried to clarify molecular characteristic in patients with different *TP53* mutation status in GC. In order to explore the genomic mechanism underling the association between *TP53* status and clinical outcomes, TCGA cohort was used for the analysis of the correlations between genomic features and TP53 status. Although tumor mutation burden (TMB) has been identified a positive predictive biomarker for better response to immunotherapy [27], there was no significant difference across subgroups based on *TP53* status (Fig. 4a), which indicated that the predictive value of *TP53* status might be independent of TMB. However, we discovered more significant instability at the chromosomal level, characterized by elevated FGA [28] (*P* < 0.001, Fig. 4b), aneuploidy score [29] (*P* < 0.001, Fig. 4c) and WGD [30] (*P* < 0.001, Fig. 4d), in *TP53* C-LOH subgroup. Moreover, patients with *TP53* C-LOH harbored a higher degree of HRD, which is one of the mechanisms of chromosome instability (*P* < 0.001, Fig. 4e, f). Consistently, *TP53* C-LOH GC were mainly enriched in CIN subtype of GC (*P* < 0.001, Fig. 4g). Together, all the above findings indicated that *TP53* C-LOH patients presented higher level of chromosomal instability.

In view of the attenuated benefit from chemotherapy and immunotherapy, we further assessed the association between *TP53* status and known targetable alterations. It was shown that *ERBB2* and *CCNE1* amplification were more significant in *TP53* C-LOH subgroup (*ERBB2* 29.21%; *CCNE1* 29.21%) compared with *TP53* CD-SC (*ERBB2* 13.83%; *CCNE1* 11.70%) and *TP53* WT subgroups (*ERBB2* 6.61%; *CCNE1* 3.08%) (Fig. 4h). Moreover, the amplification of *ERBB2* was more likely to be accompanied by *CCNE1* amplification and HRD in *TP53* C-LOH subgroup (Fig. 4i), which indicated possibility for combination of anti-HER2 therapy with PARP inhibitor or cell cycle inhibitors in these patients.

### *TP53* C-LOH promotes immune suppression in gastric cancer

**Fig. 5:**
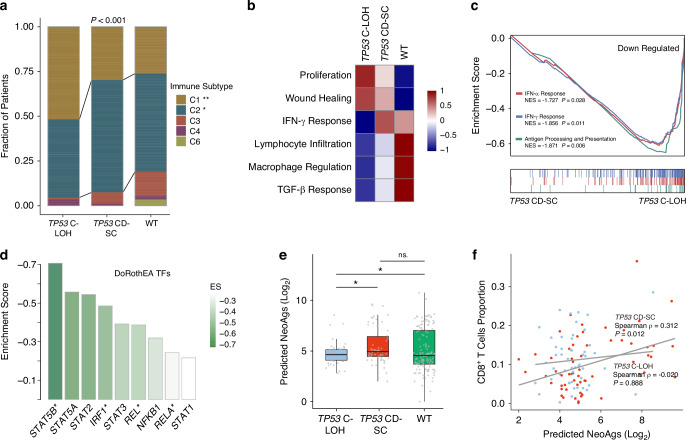
Immunophenotypic characteristics of patients with different *TP53* status. Distribution of immune subtypes across the three subgroups of interest (*TP53* C-LOH, *TP53* CD-SC and *TP53* WT) (**a**). Quantification of immune subtype signatures in the three subgroups of interest (*TP53* C-LOH, *TP53* CD-SC and *TP53* WT). **b** Gene set enrichment analysis indicated down-regulation of IFN-α Response, IFN-γ Response and Antigen Processing and Presentation pathways in *TP53* C-LOH subgroups (**c**). Enrichment analysis for immune associated transcription factor activities in *TP53* C-LOH subgroup (**d**). Boxplot for predicted neoantigens (NeoAgs) level in the three subgroups of interest (*TP53* C-LOH, *TP53* CD-SC and *TP53* WT) (**e**). The correlations of predicted neoantigens with CD8^+^ T cells in *TP53* C-LOH subgroups and *TP53* CD-SC subgroups (**f**). CD-SC clonal diploid-subclonal, C-LOH clonal with loss of heterozygosity, WT wild type.

To uncover the possible mechanisms for poor anti-PD1 immunotherapy response in *TP53* C-LOH patients, we further analyzed the immune characteristics of patients with different *TP53* mutation status in TCGA cohort and ZSHS cohort. Based on immunogenomic differences, a previous extensive analysis has identified six cancer immune subtypes: Wound Healing, IFN-γ Dominant, Inflammatory, Lymphocyte Depleted, Immunologically Quiet, and TGF-β Dominant [31]. We discovered that IFN-γ dominant phenotype was less distributed in *TP53* C-LOH subgroup (*P* < 0.001, Fig. 5a). Consistently, patients with *TP53* C-LOH showed the lowest IFN-γ Response signature (Fig. 5b). By gene set enrichment analysis, we found down-regulation of IFN-α Response, IFN-γ Response and Antigen Processing and Presentation pathways (*P* = 0.028, *P* = 0.011, *P* = 0.006; respectively; Fig. 5c). In addition, inflammatory associated transcription factors were also down-regulated in *TP53* C-LOH subgroup (Fig. 5d). Neoantigens are the basis of effective anti-tumor immune response, thus we further analyzed the association between *TP53* mutation status and tumor neoantigens predicted by computed method [32]. It was shown that patients with *TP53* C-LOH harbored significantly decreased neoantigens compared with *TP53* CD-SC and *TP53* WT patients (Fig. 5e). Interestingly, CD8^+^ T cells was not positively correlated with neoantigens in *TP53* C-LOH subgroup (Fig. 5f), and further analysis revealed that the infiltration of CD8^+^ T cells was independent of p53 status (Supplementary Fig. S4), which indicated that neoantigens might not trigger anti-tumor immune response in this subgroup of GC. Taken together, all these findings revealed that *TP53* C-LOH might promote immune suppression in GC.

## Discussion

In clinical setting, rapid advances in sequencing technology and the advent of deep genomic analysis have broadened the horizon of insight into intrinsic tumor characteristics and led to the identification of novel therapeutic targets and biomarkers [3]. Genomic alterations have been shown to be strongly associated with therapeutic responsiveness and tumor progression in various tumors, including GC [33–36]. *TP53* is the guardian of the genome, and there is a recognized association between its loss and genomic instability [37, 38]. Moreover, *TP53* mutation has been shown to be associated with patient survival risk and treatment response in several cancer types [12, 14]. However, the clinical significance of *TP53* alterations in GC has not been fully elucidated. In this study, we identified different types of *TP53* mutation based on clonality/LOH status and revealed, for the first time, that different *TP53* mutations matched with different genomic and immune microenvironment features and patient clinical outcomes.

*TP53* mutation is among the most frequent mutations in GC, affecting more than 40% of GC patients [39]. Previous studies tended to be limited to focus on the impact of overall *TP53* mutation on patient survival status, resulting in its contribution to the clinical course of the disease often being overlooked and largely underestimated. Our study in multiple independent cohorts found that compared to *TP53* WT, only *TP53* CD-SC was able to suggest better OS in GC patients, while patients with *TP53* C-LOH had similar survival outcomes as *TP53* WT patients. These results suggest that the inclusion of clonality/LOH factors in the assessment of mutational status can help to provide a more refined interpretation of high-dimensional sequencing data and mitigate the impact of background noise from synonymous mutations at the clinical level [40], thus locating the true clinically significant mutational subtypes to the extent possible.

We found that *TP53* clonal status affects the response of GC patients with *TP53* mutation to first-line treatment strategies. Patients with *TP53* CD-SC had the best responsiveness to 5-fluoropyrimidine-based ACT compared to *TP53* WT and *TP53* C-LOH patients. After receiving pembrolizumab monotherapy, *TP53* WT patients showed the optimal response, while patients with *TP53* C-LOH barely responded to ICI, suggesting that in future clinical practice, it may be possible to precisely stage GC patients based on *TP53* mutation status. In patients with *TP53* C-LOH, existing first-line treatment strategies may not be effective, and combination therapy or exploration of novel treatment strategies were required for this population, while patients with *TP53* CD-SC may receive standard ACT or ICI. Although this decision must take into account other clinicopathological factors (age, tumor status, disease burden, etc.), our study suggests that *TP53* status may help us in clinical decision making and promote individualized treatment of GC patients.

In view of the refractory nature of *TP53* C-LOH GC, we sought to further explore their genomic and immunological features in order to find the underlying mechanisms and potential therapeutic targets for their treatment insensitivity. We found that *TP53* C-LOH GC exhibited higher levels of chromosomal instability, as evidenced by elevated FGA, whole ploidy scores, whole genome duplication and HRD levels. Interestingly, *TP53* C-LOH was also strongly associated with lower neoantigen levels and inflammatory responses, suggesting a ‘cold’ immune phenotype, providing a possible explanation for the poor responsiveness to ACT and immunotherapy. In addition, the concomitant amplification of *ERBB2* and *CCNE1* observed in GC with *TP53* C-LOH, combined with its chromosomal instability, suggests that anti-HER2 therapy combined with PARP inhibitors or cell cycle inhibitors may be applied to improve the clinical outcome in this population.

Even though our results were reproducible in independent cohorts, we are aware that there still existed some limitations in our study. Inter-cohort heterogeneity must be emphasized because of the differences in epidemiology, clinicopathological characteristics, and therapeutic strategies of GC patients from the East and the West [41]. Some of the conclusions in our study were based on retrospective observations of subgroups and partially did not achieve significant consistency across all cohorts. Furthermore, the efficacy and safety of combinatory therapy for *TP53* C-LOH GC has not been assessed. Thus, further validation is required to confirm our results within the framework of more extensive, multi-centered clinical trials. Additionally, owing to data source constraints, it is challenging to evaluate the association between *TP53* mutation status and metastatic burden. Future studies focusing on cohorts of metastatic GC patients are needed to explore this relationship further.

## Supplementary information

Supplementary File

## Data Availability

Data and materials generated that are relevant to the results are included in this article. Other data are available from the corresponding author Prof. Xu upon reasonable request.

## References

[1] Levine AJ. p53: 800 million years of evolution and 40 years of discovery. Nat Rev Cancer. 2020;20:471–80. 32404993 10.1038/s41568-020-0262-1

[2] Hassin O, Oren M. Drugging p53 in cancer: one protein, many targets. Nat Rev Drug Discov. 2023;22:127–44. 36216888 10.1038/s41573-022-00571-8 PMC9549847

[3] Samstein RM, Lee CH, Shoushtari AN, Hellmann MD, Shen R, Janjigian YY, et al. Tumor mutational load predicts survival after immunotherapy across multiple cancer types. Nat Genet. 2019;51:202–6. 30643254 10.1038/s41588-018-0312-8 PMC6365097

[4] Tan X, Banerjee P, Shi L, Xiao GY, Rodriguez BL, Grzeskowiak CL, et al. p53 loss activates prometastatic secretory vesicle biogenesis in the Golgi. Sci Adv. 2021;7:eabf4885. 34144984 10.1126/sciadv.abf4885 PMC8213221

[5] Giorgi C, Bonora M, Sorrentino G, Missiroli S, Poletti F, Suski JM, et al. p53 at the endoplasmic reticulum regulates apoptosis in a Ca2+-dependent manner. Proc Natl Acad Sci USA. 2015;112:1779–84. 25624484 10.1073/pnas.1410723112 PMC4330769

[6] Vaseva AV, Moll UM. The mitochondrial p53 pathway. Biochim Biophys Acta. 2009;1787:414–20. 19007744 10.1016/j.bbabio.2008.10.005 PMC2819081

[7] Speidel D. Transcription-independent p53 apoptosis: an alternative route to death. Trends Cell Biol. 2010;20:14–24. 19879762 10.1016/j.tcb.2009.10.002

[8] Łasut-Szyszka B, Rusin M. The wheel of p53 helps to drive the immune system. Int J Mol Sci. 2023;24:7645. 37108808 10.3390/ijms24087645 PMC10143509

[9] Blagih J, Buck MD, Vousden KH. p53, cancer and the immune response. J Cell Sci. 2020;133:jcs237453. 32144194 10.1242/jcs.237453

[10] Blanchet A, Bourgmayer A, Kurtz JE, Mellitzer G, Gaiddon C. Isoforms of the p53 family and gastric cancer: a Ménage à Trois for an unfinished affair. Cancers (Basel). 2021;13:916. 33671606 10.3390/cancers13040916 PMC7926742

[11] Cancer Genome Atlas Research Network. Comprehensive molecular characterization of gastric adenocarcinoma. Nature. 2014;513:202–9. 25079317 10.1038/nature13480 PMC4170219

[12] Lim BH, Soong R, Grieu F, Robbins PD, House AK, Iacopetta BJ. p53 accumulation and mutation are prognostic indicators of poor survival in human gastric carcinoma. Int J Cancer. 1996;69:200–4. 8682588 10.1002/(SICI)1097-0215(19960621)69:3<200::AID-IJC9>3.0.CO;2-3

[13] Hamada M, Fujiwara T, Hizuta A, Gochi A, Naomoto Y, Takakura N, et al. The p53 gene is a potent determinant of chemosensitivity and radiosensitivity in gastric and colorectal cancers. J Cancer Res Clin Oncol. 1996;122:360–5. 8642047 10.1007/BF01220804 PMC12200597

[14] Bataille F, Rümmele P, Dietmaier W, Gaag D, Klebl F, Reichle A, et al. Alterations in p53 predict response to preoperative high dose chemotherapy in patients with gastric cancer. Mol Pathol. 2003;56:286–92. 14514923 10.1136/mp.56.5.286 PMC1187340

[15] Ott K, Vogelsang H, Mueller J, Becker K, Müller M, Fink U, et al. Chromosomal instability rather than p53 mutation is associated with response to neoadjuvant cisplatin-based chemotherapy in gastric carcinoma. Clin Cancer Res. 2003;9:2307–15. 12796400

[16] Hamakawa T, Kukita Y, Kurokawa Y, Miyazaki Y, Takahashi T, Yamasaki M, et al. Monitoring gastric cancer progression with circulating tumour DNA. Br J Cancer. 2015;112:352–6. 25490524 10.1038/bjc.2014.609 PMC4453461

[17] Jiang Z, Liu Z, Li M, Chen C, Wang X. Immunogenomics analysis reveals that TP53 mutations inhibit tumor immunity in gastric cancer. Transl Oncol. 2018;11:1171–87. 30059832 10.1016/j.tranon.2018.07.012 PMC6078052

[18] Kim JY, Jung J, Kim KM, Lee J, Im YH. TP53 mutations predict poor response to immunotherapy in patients with metastatic solid tumors. Cancer Med. 2023;12:12438–51. 37081749 10.1002/cam4.5953 PMC10278489

[19] Nobili S, Bruno L, Landini I, Napoli C, Bechi P, Tonelli F, et al. Genomic and genetic alterations influence the progression of gastric cancer. World J Gastroenterol. 2011;17:290–9. 21253387 10.3748/wjg.v17.i3.290 PMC3022288

[20] Sano T, Tsujino T, Yoshida K, Nakayama H, Haruma K, Ito H, et al. Frequent loss of heterozygosity on chromosomes 1q, 5q, and 17p in human gastric carcinomas. Cancer Res. 1991;51:2926–31. 2032230

[21] Datta J, Da Silva EM, Kandoth C, Song T, Russo AE, Hernandez JM, et al. Poor survival after resection of early gastric cancer: extremes of survivorship analysis reveal distinct genomic profile. Br J Surg. 2020;107:14–9. 31763684 10.1002/bjs.11443 PMC7453628

[22] Scalera S, Ricciuti B, Mazzotta M, Calonaci N, Alessi JV, Cipriani L, et al. Clonal KEAP1 mutations with loss of heterozygosity share reduced immunotherapy efficacy and low immune cell infiltration in lung adenocarcinoma. Ann Oncol. 2023;34:275–88. 36526124 10.1016/j.annonc.2022.12.002

[23] Bang YJ, Kim YW, Yang HK, Chung HC, Park YK, Lee KH, et al. Adjuvant capecitabine and oxaliplatin for gastric cancer after D2 gastrectomy (CLASSIC): a phase 3 open-label, randomised controlled trial. Lancet. 2012;379:315–21. 22226517 10.1016/S0140-6736(11)61873-4

[24] Paoletti X, Oba K, Burzykowski T, Michiels S, Ohashi Y, Pignon JP, et al. Benefit of adjuvant chemotherapy for resectable gastric cancer: a meta-analysis. Jama 2010;303:1729–37. 20442389 10.1001/jama.2010.534

[25] Mantovani F, Collavin L, Del Sal G. Mutant p53 as a guardian of the cancer cell. Cell Death Differ. 2019;26:199–212. 30538286 10.1038/s41418-018-0246-9 PMC6329812

[26] Kim ST, Cristescu R, Bass AJ, Kim KM, Odegaard JI, Kim K, et al. Comprehensive molecular characterization of clinical responses to PD-1 inhibition in metastatic gastric cancer. Nat Med. 2018;24:1449–58. 30013197 10.1038/s41591-018-0101-z

[27] Chan TA, Yarchoan M, Jaffee E, Swanton C, Quezada SA, Stenzinger A, et al. Development of tumor mutation burden as an immunotherapy biomarker: utility for the oncology clinic. Ann Oncol. 2019;30:44–56. 30395155 10.1093/annonc/mdy495 PMC6336005

[28] Sipos O, Tovey H, Quist J, Haider S, Nowinski S, Gazinska P, et al. Assessment of structural chromosomal instability phenotypes as biomarkers of carboplatin response in triple negative breast cancer: the TNT trial. Ann Oncol. 2021;32:58–65. 33098992 10.1016/j.annonc.2020.10.475 PMC7784666

[29] Vasudevan A, Schukken KM, Sausville EL, Girish V, Adebambo OA, Sheltzer JM. Aneuploidy as a promoter and suppressor of malignant growth. Nat Rev Cancer. 2021;21:89–103. 33432169 10.1038/s41568-020-00321-1

[30] Lau TY, Poon RYC. Whole-genome duplication and genome instability in cancer cells: double the trouble. Int J Mol Sci. 2023;24:3733. 36835147 10.3390/ijms24043733 PMC9959281

[31] Thorsson V, Gibbs DL, Brown SD, Wolf D, Bortone DS, Ou Yang TH, et al. The immune landscape of cancer. Immunity. 2018;48:812–30.e14 29628290 10.1016/j.immuni.2018.03.023 PMC5982584

[32] Rooney MS, Shukla SA, Wu CJ, Getz G, Hacohen N. Molecular and genetic properties of tumors associated with local immune cytolytic activity. Cell. 2015;160:48–61. 25594174 10.1016/j.cell.2014.12.033 PMC4856474

[33] Van Allen EM, Mouw KW, Kim P, Iyer G, Wagle N, Al-Ahmadie H, et al. Somatic ERCC2 mutations correlate with cisplatin sensitivity in muscle-invasive urothelial carcinoma. Cancer Discov. 2014;4:1140–53. 25096233 10.1158/2159-8290.CD-14-0623 PMC4238969

[34] Zhang C, Liu J, Xu D, Zhang T, Hu W, Feng Z. Gain-of-function mutant p53 in cancer progression and therapy. J Mol Cell Biol. 2020;12:674–87. 32722796 10.1093/jmcb/mjaa040 PMC7749743

[35] Iranzo J, Martincorena I, Koonin EV. Cancer-mutation network and the number and specificity of driver mutations. Proc Natl Acad Sci USA. 2018;115:E6010–e9. 29895694 10.1073/pnas.1803155115 PMC6042135

[36] Kandoth C, McLellan MD, Vandin F, Ye K, Niu B, Lu C, et al. Mutational landscape and significance across 12 major cancer types. Nature. 2013;502:333–9. 24132290 10.1038/nature12634 PMC3927368

[37] Baslan T, Morris JPT, Zhao Z, Reyes J, Ho YJ, Tsanov KM, et al. Ordered and deterministic cancer genome evolution after p53 loss. Nature. 2022;608:795–802. 35978189 10.1038/s41586-022-05082-5 PMC9402436

[38] Eischen CM. Genome stability requires p53. Cold Spring Harb Perspect Med. 2016;6:a026096. 27252396 10.1101/cshperspect.a026096 PMC4888814

[39] Gu Y, Zhang P, Wang J, Lin C, Liu H, Li H, et al. Somatic ARID1A mutation stratifies patients with gastric cancer to PD-1 blockade and adjuvant chemotherapy. Cancer Immunol Immunother. 2023;72:1199–208. 36369379 10.1007/s00262-022-03326-x PMC10110689

[40] Nguyen B, Fong C, Luthra A, Smith SA, DiNatale RG, Nandakumar S, et al. Genomic characterization of metastatic patterns from prospective clinical sequencing of 25,000 patients. Cell 2022;185:563–75.e11 35120664 10.1016/j.cell.2022.01.003 PMC9147702

[41] Wang FH, Zhang XT, Li YF, Tang L, Qu XJ, Ying JE, et al. The chinese society of clinical oncology (CSCO): clinical guidelines for the diagnosis and treatment of gastric cancer, 2021. Cancer Commun (Lond). 2021;41:747–95. 34197702 10.1002/cac2.12193 PMC8360643

