# Supplementary material for: Impact of clonal TP53 mutations with loss of heterozygosity on adjuvant chemotherapy and immunotherapy in gastric cancer
Source: Br J Cancer. 2024 Aug 31;131(8):1320–7. doi: 10.1038/s41416-024-02825-1 (PMC11473753; doi:10.1038/s41416-024-02825-1)
Supplement: Supplementary file 1 — Supplementary File [file 41416_2024_2825_MOESM1_ESM.docx]

**Impact of clonal *TP53* mutations with loss of heterozygosity on adjuvant chemotherapy and immunotherapy in gastric cancer**

***Running Title: TP53 C-LOH in gastric cancer***

**Key words**: gastric cancer, adjuvant chemotherapy, immunotherapy, *TP53* mutations, loss of heterozygosity.

**Supplementary Figures and Tables**

Supplementary Figure S1. Representative images of IHC and HE staining for p53 status.

Supplementary Figure S2. Correlation between TP53 status and p53 expression.

Supplementary Figure S3. Overlap test between TP53 mutation status and immunotherapy response predictors.

Supplementary Figure S4. Correlation of CD8+ T cells with p53 status.

Supplementary Table S1. Gene list to classificate TP53 C-LOH and TP53 CD-SC.

**Supplementary Figure S1. Representative images of IHC and HE staining for p53 status.** Representative images of IHC (left) and HE (right) staining for p53 WT (A), p53 Null (B) and p53 OE (C). IHC, Immunohistochemistry. HE, Hematoxylin & Eosin. WT, Wild Type. OE, Overexpression.

**
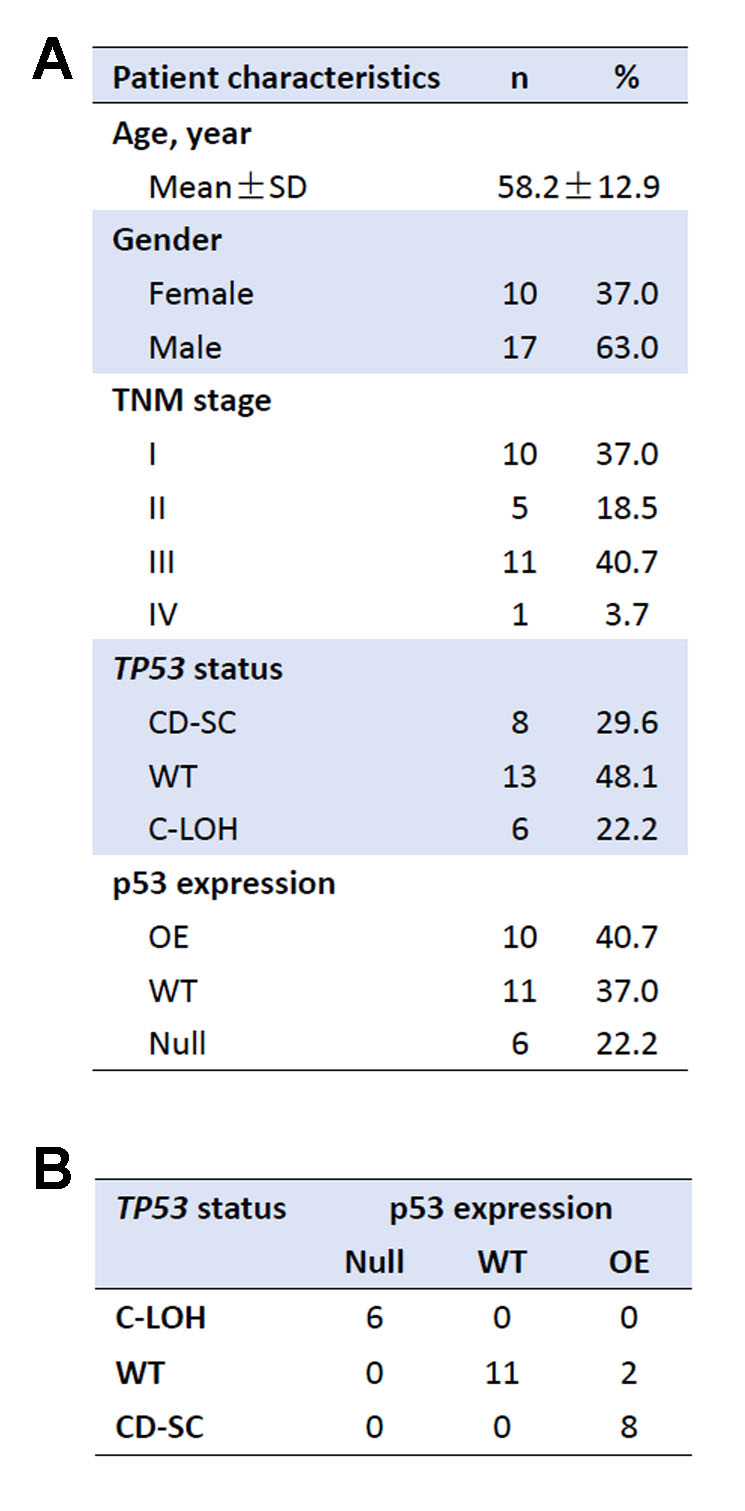
**

**Supplementary Figure S2. Correlation between TP53 status and p53 expression.** (A)The sum of patient characteristics of the FUSCC cohort. (B)The association between TP53 status (C-LOH, CD-SC and WT) and p53 expression (OE, Null and WT). C-LOH, Clonal mutations with Loss of Heterozygosity. CD-SC, Clonal diploid or subclonal mutations. WT, Wild Type. OE, Overexpression


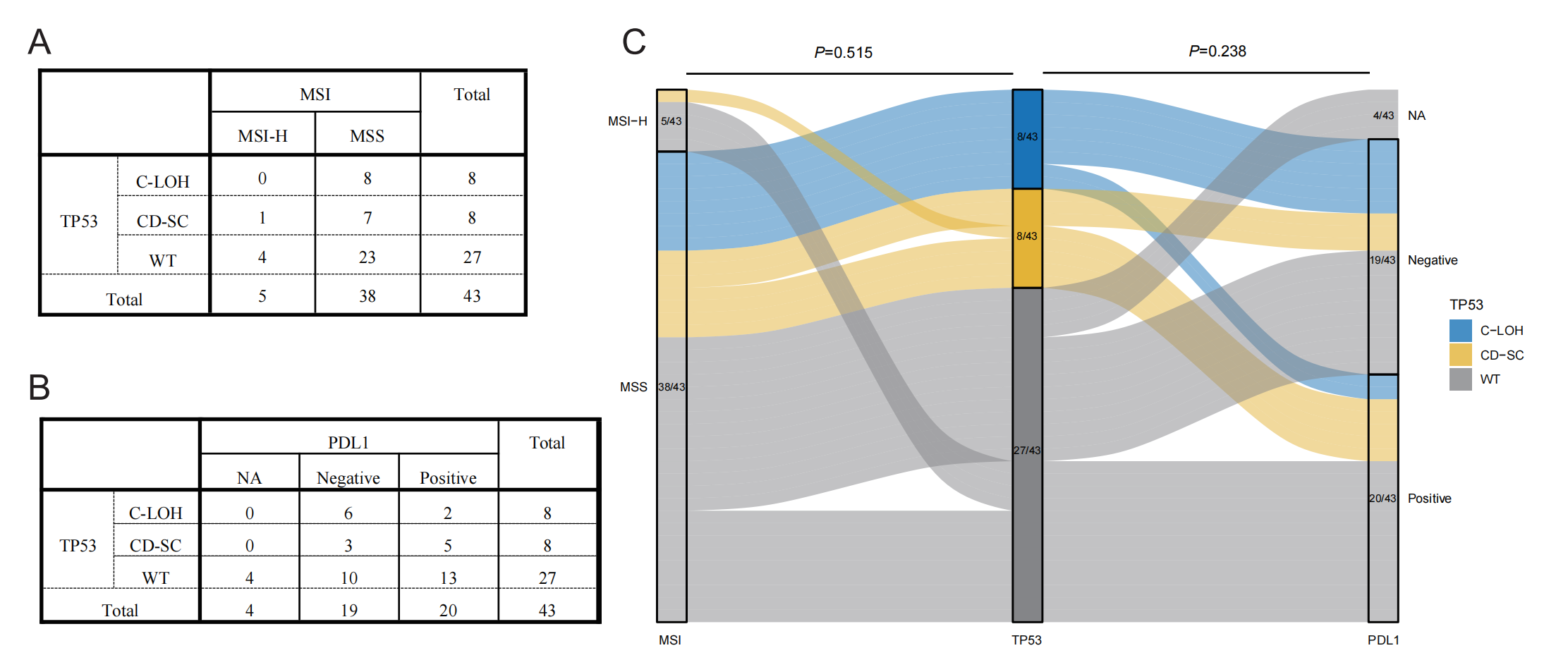


**Supplementary Figure S3. Overlap test between TP53 mutation status and immunotherapy response predictors**. (A-B) Cross-tabulations of TP53 mutation status with MSI status (A) and PD-L1 expression (B). (C) Sankey diagram demonstrating the relationship between TP53 mutation status (C-LOH, CD-SC and WT) and MSI (MSI-H and MSS), PD-L1 expression (Positive, Negative and NA). C-LOH, Clonal mutations with Loss of Heterozygosity. CD-SC, Clonal diploid or subclonal mutations. MSI-H, Microsatellite Instability - High. MSS, Microsatellite Stability. WT, Wild Type.

**Supplementary Figure S4. Correlation of CD8+ T cells with p53 status.** (A) Differences in CD8+T cells infiltration among p53 status via IHC in ZSHS cohort. (B) Differences in CXCR5+CD8+T cells infiltration among p53 status via IHC in ZSHS cohort.

**Supplementary Table S1. Gene list to classificate TP53 C-LOH and TP53 CD-SC.**

| **Supplementary Table S1. Gene list to classificate TP53 C-LOH and TP53 CD-SC** | |
| --- | --- |
| **Gene sets** | **Genes** |
| **Classification of TP53 C-LOH and TP53 CD-SC** | ANKHD1-EIF4EBP3, ARL6IP5, CALM1, COL4A3BP, DAPP1, FAS, IFI27L2, MOCS2, NDUFS4, PGGT1B, PPP2CA, REEP5, RMDN3, RNF19B, RUFY1, SQRDL, TNIP1, TRIM21, VIT, ZCCHC10, CSE1L, DDX2, DHX34, DSN1, FLVCR1, FUT1, GPT2, MAPRE1, MEX3A, MSTO2P, NKPD1, NORAD, PIGM, SERPIND1, SLC43A1, STAU1, TGIF2, TP53RK, TTI1, UBAP2L |
